# Impact of a multimodal hand hygiene improvement intervention in a 1000-bed hospital in NE Thailand: a stepped wedge clustered randomized controlled trial

**DOI:** 10.1186/2047-2994-4-S1-O19

**Published:** 2015-06-16

**Authors:** M Hongsuwan, P Srisamang, NPJ Day, D Limmathurotsakul, BS Cooper

**Affiliations:** 1 Mahidol-Oxford Tropical Medicine Research Unit, Faculty of Tropical Medicine, Mahidol University, Bangkok, Thailand; 2 Department of Pediatrics, Sappasithiprasong Hospital, Ubon Ratchatani, Thailand; 3 Centre for Tropical Medicine, Nuffield Department of Clinical Medicine, University of Oxford, Oxford, UK; 4 Department of Tropical Hygiene, Faculty of Tropical Medicine, Mahidol University, Bangkok, Thailand

## Introduction

Improving hand hygiene (HH) compliance amongst healthcare workers (HCWs) is one of the simplest and most effective measures for preventing hospital-acquired infections (HAIs). However, only a few studies have evaluated the effectiveness of interventions for improving HH compliance using strong study designs, and almost all of these have been in high income countries.

## Objectives

To evaluate the impact of a multimodal hand hygiene improvement strategy on directly observed hand hygiene compliance.

## Methods

### Design

Prospective stepped wedge randomised controlled trial using 58 in-patient hospital wards (the study clusters), and the timing of the intervention in each ward was randomly selected using a computer generated sequence.

### Setting

A 1000-bed hospital located in NE Thailand between November 2013 and April 2015.

### Intervention

The intervention was adapted from The World Health Organization’s Hand Hygiene Improvement Strategy. The intervention will be delivered by the infection control team and the infection control ward nurses who will receive additional training. A novel feature of the intervention is that staff on each ward were asked to actively decide how best to implement each of the five components of the WHO strategy. The primary analysis will be performed at the cluster level, with one observation of mean HH compliance within each ward for each time period and will use a generalized linear mixed model.

## Results

The study is still ongoing and will finish on 30^th^ April 2015. Results of the study are not available at the moment but will be presented at the meeting.

## Conclusion

To our knowledge this study will represent one of the most rigorous evaluations of the WHO Multimodal Hand Hygiene Improvement Strategy outside a high income country, and we anticipate that is should provide outcomes that enable further refinements in HH improvement strategies and that can potentially inform health economic models. Results from this study are intended to be generalisable to other resource-constrained hospitals in Thailand and elsewhere. The possibility of contamination between clusters is a potential limitation.

## Disclosure of interest

None declared.

